# Differences in fluid removal of different open-pore elements for endoscopic negative pressure therapy in the upper gastrointestinal tract

**DOI:** 10.1038/s41598-022-17700-3

**Published:** 2022-08-16

**Authors:** Kai Tobias Jansen, Jürgen Hetzel, Carola Schulte, Nurgül Düzenli, Stefano Fusco, Emanuel Zerabruck, Eva Schmider, Nisar P. Malek, Alfred Königsrainer, Dietmar Stüker, Christoph R. Werner, Dörte Wichmann

**Affiliations:** 1 Department of General, Visceral and Transplantation Surgery, University Hospital of Tübingen, Hoppe-Seyler-Str. 3, 72076 Tübingen, Germany; 2 Department of Internal Medicine VIII, Pulmonology and Oncology, University Hospital of Tübingen, Otfried-Müller-Str. 10, 72076 Tübingen, Germany; 3 Devision of Pulmonology, Department of Internal Medicine, Cantonal Hospital Winterthur, Brauer-Straße 15, 8400 Winterthur, Switzerland; 4 Department of Internal Medicine I, Gastroenterology, Hepatology, Gastrointestinal Oncology, Infectiology and Geriatrics, University Hospital of Tübingen, Otfried-Mueller-Str. 10, 72076 Tübingen, Germany; 5 Interdisciplinary Endoscopic Unit, University Hospital of Tübingen, Hoppe-Seyler-Str. 6, 72076 Tübingen, Germany

**Keywords:** Oesophagogastroscopy, Gastroenterology, Oesophageal diseases

## Abstract

Endoscopic negative pressure therapy is an effective treatment strategy for various defects of the gastrointestinal tract. The functional principle is based on an open-pore element, which is placed around a perforated drainage tube and connected to a vacuum source. The resulting open-pore suction device can undergo endoluminal or intracavitary placement. Different open-pore suction devices are used for endoscopic negative pressure therapy of upper gastrointestinal tract defects. Comparative analyses for features and properties of these devices are still lacking. Eight different (six hand-made devices and two commercial devices) open-pore suction devices for endoscopic negative pressure therapy of the upper gastrointestinal tract were used, amount fluid removed was evaluated. The evaluation parameters included the time to reach the target pressure, the time required to remove 100 ml of water, and the material resistance of the device. All open-pore suction devices are able to aspirate the target volume of fluids. The time to reach the target volume varied considerably. Target negative pressure was not achieved with all open-pore suction devices during the aspiration of fluids; however, there was no negative effect on suction efficiency. Of the measurement data, material resistance could be calculated for six open-pore elements. We present a simple experimental, nonphysiologically setup for open-pore suction devices used for endoscopic negative pressure therapy. The expected quantity of fluids secreted into the treated organs should affect open-pore suction device for endoscopic negative pressure therapy.

## Introduction

In 2000, endoscopic negative pressure therapy (ENPT) was introduced by Weidenhagen and Gruetzner to address the insufficiencies of rectal anastomoses. An open-pore polyurethane (PU) sponge was placed into the perianastomotic cavity. The sponge was attached to a perforated drainage tube connected to a vacuum source. The success of this technique was primarily presented as conference contributions (for example: 46. Austrian Congress of Surgeons, Weidenhagen et al. 2004; 64. Congress of the German Society of Gastroenterology (DGVS), Loske G and Weidenhagen R. 2009).

The first case series of ENPT was published in 2006^1^. Due to the evident safety and efficacy of this treatment mode, ENPT was used to treat other perforating defects of the gastrointestinal tract. Its applications for the UGI were reported since 2007^2–5^. ENPT improves local perfusion, resolves interstitial wound oedema, removes fluids, and debrides the wound base. Vital granulation tissue is formed after wound cleaning^6^. ENPT is also named Endovac therapy and endoscopic vacuum Therapy (EVAC or EVT).

The basic principle of ENPT is an open-pore element sheathing the perforated distal end of a tube, and resulting product is called open-pore suction device (OPSD). From 2000 until 2015, PU sponges were exclusively used for ENPT. Then, the CNP drainage-film was introduced by Loske to establish ENPT in the urinary and gastrointestinal tracts^7,8^. The CNP film is an open-pore drainage film that can be used to create a thin ENPT device through wrapping on gastrointestinal or Redon tubes. Simultaneous ENPT and tube feeding of patients is possible using two- or three-lumen feeding tubes. In the article “Tips and tricks for endoscopic negative pressure therapy” Loske introduced different OPSDs based on the open-pore film and PU sponge^9^. In 2018, Heiss introduced a new device for ENPT of the UGI that combines the benefits of self-expandable metal stent-therapy and ENPT: the VAC Stent^10^. The advantages of this stent are the possibility of oral food intake in combined with continuous wound cleaning by EPNT.

We have implemented ENPT as first-line treatment strategy for various defects in the UGI since 2017^11,12^. The OPSD used for different locations and indications is chosen by the endoscopist according to his or her expertise and preferences. An analysis of the characteristics of OPSDs are still lacking.

## Results

### Achievement of the target negative pressure

The target negative pressure was achieved in 18 of 24 measurements. In three tests with prototype gastric tubes with CNP film (a), a target vacuum was achieved. In tests with the Trelumina drainage with CNP-film wrapped gastric tube (d), the target negative pressure was achieved in two of three measurements before 100 ml water was removed.

### Time to achievement of the target vacuum level

The two commercially available OPSDs (Eso-Sponge g = 27.2 s; VACS h = 28.3 s) and the enteral feeding tubes wrapped with a PU-sponge and CNP-film (f = 13.2 s) allow rapid achievement of 125 mmHg or 16,665.25 Pa suction.

### Removal of 100 ml

One hundred millilitres of water were removed for every measurement with every OPSD. The mean evacuation time for all tests was 115.94 s. The suction time differed considerably between the different OPSDs. Fast evacuation of 100 ml water was achieved by three prototype OPSDs (a = mean 26.09 s; b = mean 29.43 s; d = mean 26.43 s). Of the commercially available ENPT products, the evacuation times of the target amount of water were similar (g = mean 57.4 s; h = mean 53.45 s). The prototype enteral feeding tube with PU-sponge and CNP-film (f = mean 327.5 s) achieved the longest time for removal of 100 ml water.

### Calculation of material resistance

Material resistance was calculated with the following formula: R = U/I, with U is ∆ pressure and I is the flow rate (volume/time). Material resistance was determined in 7 of 8 OPSDs. The calculation of resistance was possible only in cases where the given pressure of 125 mmHg was reached. Results of the measurements are summarized in Table 1.

**Table 1:** Results of measurements according to the tubes and devices for endoscopic negative pressure therapy of the upper gastrointestinal tract.

Device	Achievement of 125 mmHg negative pressure	Mean time to achievement of 125 mmHg negative pressure [sec (min;max)]	Mean time of removal of 100 ml water [sec (min, max)]	Material resistance [Δ kPa/(mL/s)]
a	No	n.a	26.1 (24.1; 27.1)	n.a
b	Yes	44.35	250.9 (25.3;390,6)	31.3
c	Yes	28.9 (5.4; 41.9)	45.8 (53.93; 59,87)	57.3
d	Yes	59.2 (58.8; 59.6)	26.4 (26.3; 26.5)	33.0
e	Yes	67.7 (7.5; 21.7)	29.4 (23.6; 40.1)	29.5
f	Yes	13.2 (17.8; 149.8)	327.5 (313.5; 336.7)	409.3
g	Yes	27.2 (5.6; 39.4)	57.4 (44.3; 65.6)	71.6
h	Yes	28.3 (27.3; 29.42)	53.5 (53.3; 53.6)	66.8

## Discussion

We present the results of a simple experimental setup for testing different OPSDs used for ENPT in the UGI. This nonphysiologically test can be used to analyse the characteristics and competencies of fluid removal. Of course, a container filled with water does not simulate the environment of the UGI, and water is not equivalent to gastrointestinal fluids. This simple test was performed to analyse modes of action of the presented OPSDs. In particular, for OPSDs placed into the duodenum for perforation therapy, a relevant quantity of fluids must be moved through the devices. To our knowledge, this is the first description of an experimental comparison of the characteristics of different OPSDs for use as ENPT devices. We are aware of the simplicity of the experimental design presented. The results for the negative pressure achievement and material resistance calculation shows the following. For tubes (16Ch) wrapped with CNP-film or PU-sponge material (probes a, d, e) rapid fluid removal is possible, but the target negative pressure is not achieved, and it is impossible to calculate the material resistance of probes a and e. The commercially available products (g and h) show mostly consistent results with similar findings in the calculation of material resistance. The self-made devices using the 16Ch nasogastric tube, intestinal tube and PU-sponge with (f) or without (b) wrapping with CNP-film resulted in the highest material resistance values and similar times to achievement of the target pressure. Time it took to remove 100 ml fluids was up to 7 min in these probes.

What do these tests tell us? The choice of OPSD for ENPT in the UGI should be made depend on the expected amount of fluids in the treated organ. Higher quantities of fluids are expected for ENPT of duodenal leakages or insufficiencies. The considerable differences between the OPSDs suggest that different treatment goals of the ENPT could be reached. A “one fits all” approach is also unlikely in the ENPT subject area.

The commercially available products for ENPT produced uniform results in the tests performed. Both analysed products are approved for ENPT in the oesophagus. Self-made OPSDs with more than two components showed variable outcomes, with increasing material resistance depending on the number of materials used.

Literature on ENPT of defects of the UGI consists of case reports and monocentric case series^13–15^, comparisons of negative pressure and stent-based therapy^16–18^, and descriptions of new opportunities of use^19,20^. We need more data about features and properties of different OPSDs used for ENPT. Points of interests are the grade of granulation induction, the optimal pressure level and the optimal treatment time in patients with defects of the UGI. To answer these key questions, we need animal models and standardised protocols. Prospective multicenter studies are desirable.

### Conclusion

Knowledge about properties and features of OPSD for ENPT is still limited. The expected quantity of fluid drainage from the treated organ, the expertise of the endoscopist, and the wound conditions should dedicate the choice of OPSD for ENPT.

## Material and methods

### Experimental setup

**Figure 1:**
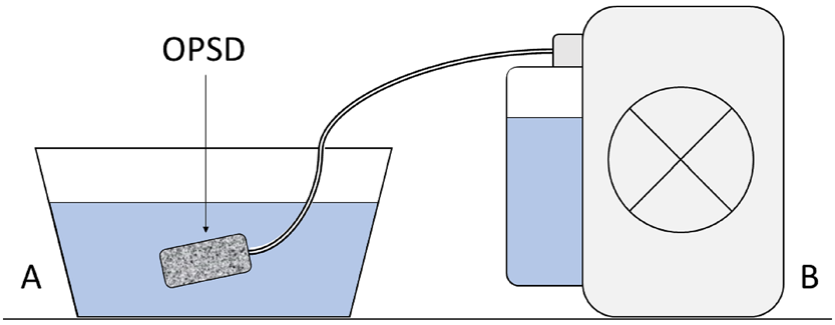
Experimental setup. (**A**) Water container, (**B**) Electric vacuum pump with water canister, OPSD: Open-pore suction device (for tested OPSD see Fig. 2).

OPSDs were individually placed into a container filled with water and were connected to an electric vacuum pump with pressure monitoring (KCI V.A.C. Ultra Therapy Unit, KCI USA Inc., San Antonio, Texas, United States, see Fig. 1). The pressure of the target vacuum was 125 mmHg in all measurements according to the negative pressure usually used for ENPT of the gastrointestinal tract^9,12,16^. The measured values were as follows: the achievement of the target negative pressure, the time taken to reach the negative pressure and the time taken to aspirate 100 ml water.

**Figure 2:**
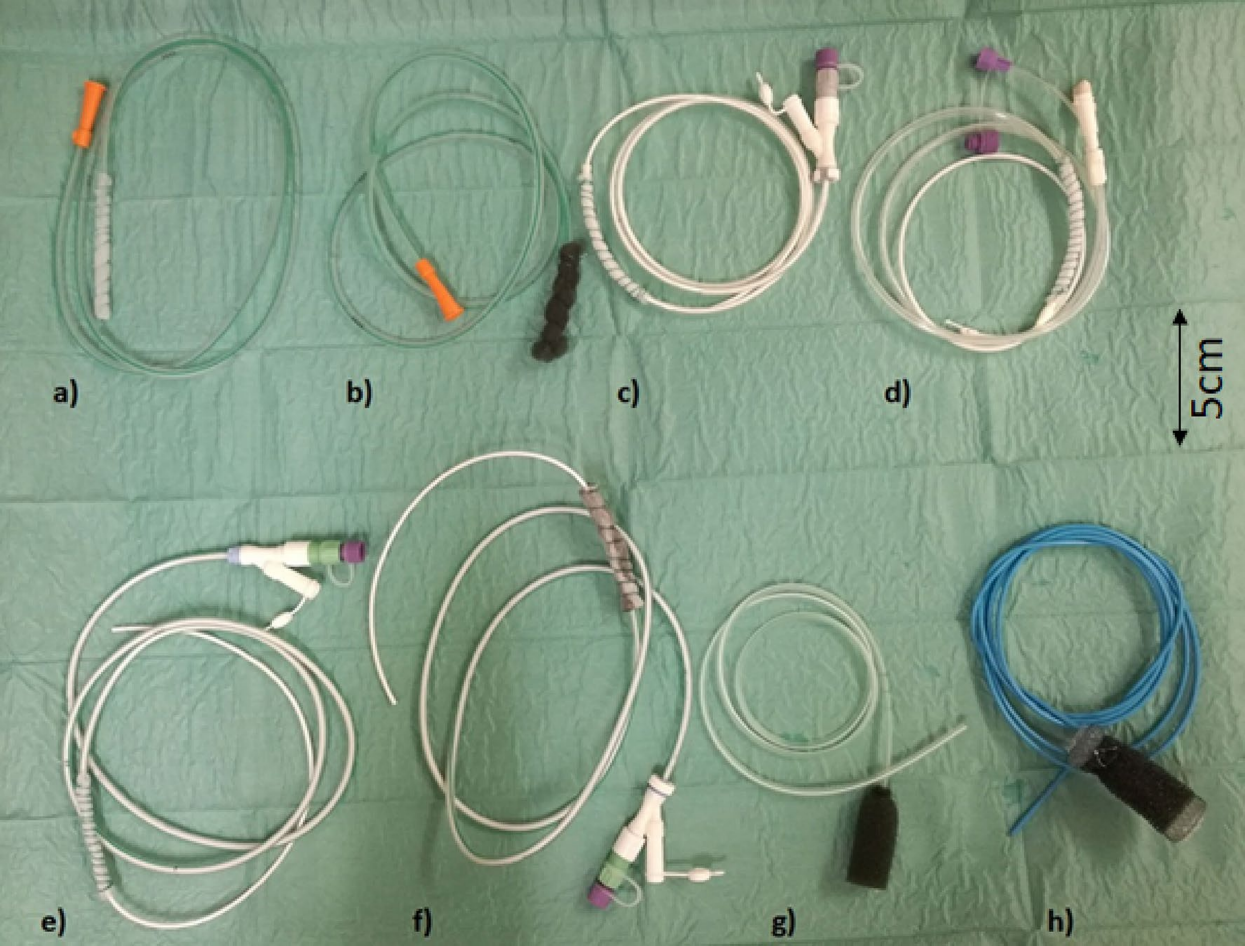
Tested open-pore suction devices for ENPT of the UGIT. (**a**) Self-made gastric tube with CNP-film wrapped around the distal perforations. (**b**) Self-made PU sponge drainage on a 16Ch nasogastric tube. (**c**) Enteral feeding tube with two lumen and wrapped CNP-film around the gastric tube. (**d**) Enteral feeding tube with three lumen and wrapped CNP-film around the gastric tube. (**e**) Self-made feeding tube, using a 16Ch nasojejunal tube (length 125 cm) with an introduced 9Ch intestinal tube and CNP-film around the gastric tube. (**f**) Self-made combination of two OPSD. The distal end of a nasogastric tube (16Ch) is wrapped with a PU sponge. Over the sponge the CNP-film is single-layered placed. (**g**) The commercially available Eso-Sponge system a PU sponge with a fixed 9Ch drainage tube. (**h**) The new commercially available combination product VAC-Stent with integrated 9Ch drainage tube. ENPT: endoscopic negative pressure therapy; OPSD: open-pore suction device; PU: polyurethane; UGIT: upper gastrointestinal tract.

Eight open-pore suction devices were tested (Fig. 2). Every probe was tested three times. Differences and advantages of the different prototype and commercial OPSDs are summarised in Table 2.

**Table 2:** Characteristics and clinical features of the different analysed OPSDs.

Device	Short-description of the device	Benefits	Limitations	EL or IC position	Changing interval
a	Self-made OPSD with drainage film on gastric tube	Simple to create and to place, thin, good fluid removal	No feeding option	EL + IC	Up to 7 days
b	Self-made OPSD with PU-sponge on gastric tube	Simple to create and to place, good cleaning and tissue growth effect	No feeding option	EL + IC	3–5 days
c	Self-made OPSD with drainage film on gastric tube of a two-lumen tube	Simple to create and to place, thin, good fluid removal, feeding offer through intestinal tube with variable length	Gastric part with pre-fabricated length	EL	Up to 7 days
d	Self-made OPSD with drainage film on gastric part of a treluminal feeding tube	Simple to create and to place, thin, good fluid removal, feeding offer through intestinal tube	Gastric part and intestinal tube with pre-fabricated length	EL	Up to 7 days
e	Self-made OPSD with drainage film on duodenal tube with inserted jejunal tube	Simple to create and to place, thin, feeding offer through intestinal tube with variable length, good fluid removal, reaching duodenal leakage possible	Demanding placement	EL	Up to 7 days
f	Self-made OPSD with PU-sponge and drainage film on gastric tube with inserted jejunal tube	Little ingrowth, feeding offer through intestinal tube with variable length	Demanding to create and to place	EL	Up to 7 days
g	Commercially available Eso-SPONGE	Ready to use, easy to place	No simultaneous feeding option	EL + IC	3–5 days
h	Commercially available VACStent	Oral feeding possible, good tissue growth and cleaning	Demanding to place and to remove	EL	Up to 7 days

PU = polyurethane, EL = endoluminal, IC = intracavitary.

For the Trelumina probe (d) the ventilation tube was closed with plasters. The intestinal tubes were closed by clamps during the tests.

### Description and preparation of the used OPSDs

- A stomach tube (16 Ch, Dahlhausen, Petershagen, Germany) was wrapped with an open-pore double-layered drainage film (Suprasorb; CNP Drainage Film; Lohmann & Rauscher International GmbH & Co KG, Rengsdorf, Germany) around the distal and preperforated end. The wrapping was attached with sewing material (Mersilene, Polyester, 4 Ph. Eur; Ethicon—Johnson & Johnson Medical N.V., Belgium) by piercing the tube in the distal and proximal edge of the drainage film and wrapping the suture around the drainage film and the tube.
- A prototype PU sponge drainage on a 16Ch nasogastric tube. The PU sponge (V.A.C. Granufoam Dressing, 3 M + KCI, Texas, United States) was cut to size and placed on the distal end of a nasogastric tube (stomach tube 16 Ch, Dahlhausen, Petershagen, Germany). To ensure the position, a suture (Mersilene, Polyester, 4 Ph. Eur; Ethicon—Johnson & Johnson Medical N.V., Belgium) is wrapped around the sponge and the tube with holding sutures in the distal and proximal position.
- An enteral feeding tube with two lumens (Easyin Freka, Fresenius Kabi Deutschland GmbH, Bad Homburg, Germany), one intestinal, white feeding tube with 9 Ch and one gastric, decompression tube with 16 Ch, is wrapped with CNP-film (Suprasorb CNP Drainage Film, Lohmann & Rauscher International GmbH & Co.KG, Rengsdorf, Germany) around the pre-perforated 16 Ch decompression tube. To ensure the position of the drainage film, a suture (Mersilene, Polyester, 4 Ph. Eur; Ethicon—Johnson & Johnson Medical N.V., Belgium) was wrapped around the drainage film and the tube without piercing the tube (see a video of preparation in https://doi.org/10.1016/j.vgie.2020.10.009).
- An enteral feeding tube with three lumens (Freka Trelumina, Fresenius Kabi Deutschland GmbH, Bad Homburg, Germany) was wrapped with cut-to size CNP-film (CNP-film = Suprasorb CNP Drainage Film; Lohmann & Rauscher International GmbH & Co.KG, Rengsdorf, Germany) around the distal stomach tube with perforations. To ensure the position a suture (Mersilene, Polyester, 4 Ph. Eur; Ethicon—Johnson & Johnson Medical N.V., Belgium) was wrapped around the drainage film and the tube with holding sutures in the distal and proximal position.
- A prototype feeding tube was made using a 16Ch nasojejunal tube (Duodenal Tube Levin Dahlhausen, length 125 cm, Petershagen, Germany) and an introduced 9Ch intestinal tube (Easyin Freka, Fresenius Kabi Deutschland GmbH, Bad Homburg, Germany, Y-piece and adapter are included in the pack). Cut-to-size CNP-film (CNP-film = Suprasorb CNP Drainage Film; Lohmann & Rauscher International GmbH & Co.KG, Rengsdorf, Germany) is wrapped around the preperforated stomach tube. To ensure the position of the drainage film, a suture (Mersilene, Polyester, 4 Ph. Eur; Ethicon—Johnson & Johnson Medical N.V., Belgium) was wrapped around the drainage film and the tube without piercing the tube.
- A prototype combination of two OPSDs was made according to the description by Loske et al.^9^. The distal end of a nasogastric tube (16Ch, stomach tube 16 Ch, Dahlhausen, Petershagen, Germany) was wrapped with a cut-to-size polyurethane sponge (PU sponge = V.A.C. Granufoam Dressing, 3 M + KCI, Texas, United States). Over the sponge the CNP-film was placed single-layered (CNP-film = Suprasorb CNP Drainage Film, Lohmann & Rauscher International GmbH & Co.KG, Rengsdorf, Germany) and fixed with seam material (Mersilene, Polyester, 4 Ph. Eur; Ethicon—Johnson & Johnson Medical N.V., Belgium) as described before. An intestinal tube (9Ch, Easyin Freka, Fresenius Kabi Deutschland GmbH, Bad Homburg, Germany, Y-piece with adapter is part of the package) was inserted into the stomach tube and was pushed far beyond this.
- The commercially available Eso-Sponge system, a PU sponge with a fixed 9Ch drainage tube (Eso-Sponge, B. Braun Melsungen AG, Melsungen, Germany).
- The new commercially available combination product VACS (Vakuum-Stent or VAC-Stent, MöllerMedical GmbH, Fulda, Germany) with integrated 9Ch drainage tube.

### Sample size

No data for the features of different OPSDs are reported. Each of the OPSD (a-f) was tested three times. In sum 24 measurements were performed.

### Statistics

Data were analysed with Excel (MS Office 2019) and SPSS (IBM 2021). Data are presented in numbers and means.

## Data Availability

The datasets used and/or analysed during the current study available from the corresponding author on reasonable request.

## Abbreviations

ENPT: Endoscopic negative pressure therapy
EVAC: Endovac-therapy
EVT: Endoscopic vacuum therapy
OPSD: Open-pore suction device
PU: Polyurethane
UGIT: Upper gastrointestinal tract

## References

[1] Nagell CF, Holte K (2006). Treatment of anastomotic leakage after rectal resection with transrectal vacuum-assisted drainage (VAC). A method for rapid control of pelvic sepsis and healing. Int. J. Colorectal. Dis..

[2] Mennigen R (2013). Endoscopic closure of postoperative gastrointestinal leakages and fistulas with the Over-the-Scope Clip (OTSC). J. Gastrointest. Surg..

[3] Loske G, Muller C (2009). Vacuum therapy of an esophageal anastomotic leakage–a case report. Zentralbl. Chir..

[4] Loske G, Muller C (2009). Endoscopic vacuum-assisted closure of upper intestinal anastomotic leaks. Gastrointest. Endosc..

[5] Loske G, Schorsch T, Mueller CT (2010). Endoscopic intraluminal vacuum therapy of duodenal perforation. Endoscopy.

[6] Loske G (2018). Endoscopic negative pressure therapy of the upper gastrointestinal tract. German version. Chirurg.

[7] Loske G (2015). Successful endoscopic vacuum therapy with new open-pore film drainage in a case of iatrogenic duodenal perforation during ERCP. Endoscopy.

[8] Loske G (2017). First report of urinary endoscopic vacuum therapy : For large bladder defect after abdomino-perineal excision of the rectum. Video paper. Chirurg.

[9] Loske G, Muller CT (2018). Tips and tricks for endoscopic negative pressure therapy. German version. Chirurg.

[10] Chon SH (2020). VACStent: a new option for endoscopic vacuum therapy in patients with esophageal anastomotic leaks after upper gastrointestinal surgery. Endoscopy.

[11] Archid R (2020). Endoscopic vacuum therapy for staple line leaks after sleeve gastrectomy. Obes Surg.

[12] Wichmann D (2020). Endoscopic negative pressure therapy with open-pore film drainage and open-pore polyurethane sponge drainage for iatrogenic perforation of the esophagus. Endoscopy.

[13] Bludau M (2018). Results of endoscopic vacuum-assisted closure device for treatment of upper GI leaks. Surg. Endosc..

[14] Kuehn F (2017). Endoscopic vacuum therapy for various defects of the upper gastrointestinal tract. Surg. Endosc..

[15] Laukoetter MG (2017). Successful closure of defects in the upper gastrointestinal tract by endoscopic vacuum therapy (EVT): a prospective cohort study. Surg. Endosc..

[16] Berlth F (2019). Self-expanding metal stents versus endoscopic vacuum therapy in anastomotic leak treatment after oncologic gastroesophageal surgery. J. Gastrointest. Surg..

[17] Rausa, E., et al., *Comparison of endoscopic vacuum therapy versus endoscopic stenting for esophageal leaks: systematic review and meta-analysis.* Dis Esophagus, 2018. **31**(11). 10.1093/dote/doy060 29939229

[18] Mennigen R (2015). Comparison of endoscopic vacuum therapy versus stent for anastomotic leak after esophagectomy. J. Gastrointest. Surg..

[19] Kantowski M, Kunze A (2018). New strategies and materials in endoscopic vacuum therapy in the lower gastrointestinal tract. Chirurg.

[20] Rucktaeschel F (2019). Gastroduodenal anastomotic insufficiency: pull-through technique for endoscopic negative pressure therapy with new types of open-pore drains. Endoscopy.

